# How do you manage ANTICOagulant therapy in neurosurgery? The ANTICO survey of the Italian Society of Neurosurgery (SINCH)

**DOI:** 10.1186/s12883-021-02126-7

**Published:** 2021-03-03

**Authors:** Alessandro Prior, Pietro Fiaschi, Corrado Iaccarino, Roberto Stefini, Denise Battaglini, Alberto Balestrino, Pasquale Anania, Enrico Prior, Gianluigi Zona

**Affiliations:** 1 Section of Neurosurgery, Department of Neuroscience (DINOGMI) IRCCS Ospedale Policlinico San Martino, Genoa, Italy; 2 Università di Genova, Dipartimento di Neuroscienze, Riabilitazione, Oftalmologia, Genetica e Scienze materno infantili (DINOGMI), IRCCS Ospedale Policlinico San Martino, Largo Rosanna Benzi, 1016132 Genoa, Italy; 3 Department of Neurosurgery, University Hospital, Parma, Italy; 4 Department of Neurosurgery, Ospedale Civile di Legnano, Milan, Italy; 5 Anesthesia and Intensive Care, Policlinico San Martino Hospital, IRCCS for Oncology and Neuroscience, Genoa, Italy; 6 Department of Medicine, University of Barcelona, Barcelona, Spain; 7 Division of Cardiology, Department of Medicine University of Verona, Verona, Italy

**Keywords:** Anticoagulant, Reversal therapy, Neurosurgery, Traumatic brain injury, Spontaneous intracerebral hemorrhage

## Abstract

**Background:**

Anticoagulant assumption is a concern in neurosurgical patient that implies a delicate balance between the risk of thromboembolism versus the risk of peri- and postoperative hemorrhage.

**Methods:**

We performed a survey among 129 different neurosurgical departments in Italy to evaluate practice patterns regarding the management of neurosurgical patients taking anticoagulant drugs. Furthermore, we reviewed the available literature, with the aim of providing a comprehensive but practical summary of current recommendations.

**Results:**

Our survey revealed that there is a lack of knowledge, mostly regarding the indication and the strategies of anticoagulant reversal in neurosurgical clinical practice. This may be due a lack of national and international guidelines for the care of anticoagulated neurosurgical patients, along with the fact that coagulation and hemostasis are not simple topics for a neurosurgeon.

**Conclusions:**

To overcome this issue, establishment of hospital-wide policy concerning management of anticoagulated patients and developed in an interdisciplinary manner are strongly recommended.

**Supplementary Information:**

The online version contains supplementary material available at 10.1186/s12883-021-02126-7.

## Background

Surgical population has increasing medical complexity, with special regards to the implemented use of antiplatelets agents and anticoagulants for the prevention of thrombotic events in selected patients [1]. Neurosurgical procedures are classified as very high hemorrhagic surgical procedures, making the management of anticoagulation in neurosurgery one of the toughest challenges. The Food and Drug Administration (USA) estimated that 2 millions of people each year start using vitamin K antagonist. The use of anticoagulants is expected to continue growing in the near future [2, 3]. In recent years, direct-acting anticoagulants (DOACs) showed a safer profile when compared to vitamin K antagonists (VKAs), both in term of pharmacokinetics and reduced necessity of periodical blood monitoring, thus becoming the first choice in several cases. In neurosurgery, the implemented use of DOACs reduced the rate of spontaneous bleeding complications [4]. Otherwise, the lack of selective reversal drugs and specific test for adequate monitoring confirmed the existence of several limitations even for these novel anticoagulant drugs [4].

The use of anticoagulants is of extreme interest both in the setting of acute traumatic brain injury (TBI) or intracerebral hemorrhage (ICH) and in case of elective surgery, complicating the maintenance of the delicate balance between thromboembolic and hemorrhagic events [5]. In 2015, an update from the French Working Group on Perioperative Hemostasis (GIHP) suggested an interruption time of DOACs up to 5 days in case of elective neurosurgical procedures in the absence of renal disease, while longer in case of kidney injury and elderly age [6]. Moreover, recent recommendations on the management of anticoagulants and reversal therapy are mostly managed in medical practice and published in cardio-vascular journals, although neurosurgeons must deal with coagulation-related issues almost daily in clinical practice. Nevertheless, comprehensive guidelines on how to manage anticoagulated neurosurgical patients are lacking.

We therefore performed a national survey in Italy to depict current clinical practice for the management of anticoagulation in neurosurgical patients. Furthermore, we reviewed the available literature, with the aim of providing a comprehensive and practical summary of current recommendations.

## Methods

### Survey preparation

This national survey was endorsed by the Italian Society of Neurosurgery (SINCH) and promoted by the Department of Neuroscience, Rehabilitation, Ophthalmology, Genetics and Maternal and Child Health (DINOGMI), University of Genoa, Italy. The questionnaire was created by using an electronic software (SurveyMonkey®). With the aid of the bureau of the SINCH, a mailing list including the e-mail addresses of all the chiefs of different neurosurgical departments in Italy for a total number of 129 participants was extracted. All participants received a hyperlink for participating to the Survey. The link was sent to the mailing list in September 2017 and remained active until November 2017.

The survey consisted in 20 questions regarding the management of hemostasis, antiplatelet therapies and anticoagulation as experienced in neurosurgical care practice. The questionnaire was developed by a panel of expert neurosurgeons and hematologists highly experienced in coagulation disorders.

Ten questions regarded the use of antiplatelet therapy (previously discussed by our group [7]), while ten questions regarded the use of anticoagulants, as discussed in the current paper. Table 1 summarizes the questions raised in our Survey.

**Table 1:** The survey

***Question 1:*** ***In the last year, indicate the approximate percentage of patients admitted in your department with “acute” neurosurgical indication (***e.g. ***acute/chronic epidural-subdural hematomas, ICH, SAH, Traumatic Subarachnoid Hemorrhage), in anticoagulant therapy.***	
*Possible answers*	
o *less than 10%*	
o *between 10 and 25%*	
o *between 25 and 50%*	
o *between 50 and 75%*	
o *more than 75%.*	
***Question 2:*** ***In the clinical scenario of the above mentioned acute neurosurgical pathologies, in which conditions do you apply a “forced” emergency reversal of anticoagulant agents?***	
*Possible answers:*	
o *Only if a surgical treatment is plenned*	
o *Also If a conservative treatment is planned*	
***Question 3:*** ***In the eventuality of the clinical scenario of the above mentioned “acute neurosurgical pathologies”, how do you reverse VKA patients? For every option listened, please choose among routinely, frequently, rarely and never***	
***Vitamin K***	
o *routinely*	
o *frequently*	
o *rarely*	
o *never*	
***Vitamin K plus Fresh Frozen Plasma***	
*routinely*	
o *frequently*	
o *rarely*	
o *never*	
***Vitamin K plus Prothrombin Complex Concentrate***	
o *routinely*	
o *frequently*	
o *rarely*	
o *never*	
***Prothrombin Complex Concentrate alone***	
o *routinely*	
o *frequently*	
o *rarely*	
o *never*	
***Recombinant Activated Factor VII***	
o *routinely*	
o *frequently*	
o *rarely*	
o *never*	
***Recombinant Activated Factor VII plus Vitamin K***	
o *routinely*	
o *frequently*	
o *rarely*	
o *never*	
***Question 4:*** ***In the eventuality of the clinical scenario of the above mentioned (“acute neurosurgical pathologies”), how do you reverse DOAC patients? For every option listened, please choose among routinely, frequently, rarely and never***	
***Vitamin K***	
o *routinely*	
o *frequently*	
o *rarely*	
o *never*	
***Vitamin K plus Fresh Frozen Plasma***	
*routinely*	
o *frequently*	
o *rarely*	
o *never*	
***Vitamin K plus Prothrombin Complex Concentrate***	
o *routinely*	
o *frequently*	
o *rarely*	
o *never*	
***Prothrombin Complex Concentrate alone***	
o *routinely*	
o *frequently*	
o *rarely*	
o *never*	
***Activated Prothrombin Complex Concentrate***	
o *routinely*	
o *frequently*	
o *rarely*	
o *never*	
***Recombinant Activated Factor VII***	
o *routinely*	
o *frequently*	
o *rarely*	
o *never*	
***Recombinant Activated Factor VII plus Vitamin K***	
o *routinely*	
o *frequently*	
o *rarely*	
o *never*	
***Specific Reversal Agent (if available)***	
o *routinely*	
o *frequently*	
o *rarely*	
o *never*	
***Question 5:*** ***How do you assess anticoagulant effects in patients on DOACSs with acute neurosurgical pathologies? (multiple answers possible)***	
o *Drug’s half-life*	
o *Time from the last intake of the drug*	
o *PT/aPTT*	
o *INR*	
o *Specific assay*	
***Question 6:*** ***What is the optimal timing for initiating venous thromboembolism chemoprophylaxis after intracranial bleeding or after elective surgery?***	
o *less than 2 days*	
o *between 2 and 4 days*	
o *between 4 and 7 days*	
o *more than 7 days*	
***Question 7:*** ***In your opinion, what is the optimal timing for anti-thrombotic therapy resumption in patients at high thrombotic risk (*e.g.** ***valvular atrial fibrillation, ventricular devices)?***	
o *less than 5 days*	
o *between 5 and 10 days*	
o *more than 10 days*	
***Question 8:*** ***In your opinion, what is the optimal timing for anti-thrombotic therapy resumption in patients at moderate thrombotic risk (*e.g.** ***non-valvular atrial fibrillation)?***	
o *less than 5 days*	
o *between 5 and 10 days*	
o *more than 10 days*	
***Question 9:*** ***In your opinion, what is the optimal timing for anti-thrombotic therapy resumption in patients atlow-thrombotic risk (*e.g.** ***previous history of deep venous thrombosis)?***	
o *less than 5 days*	
o *between 5 and 10 days*	
o *more than 10 days*	
***Question 10:*** ***Do you usually ask for a cardiological evaluation for the perioperative management of anticoagulated patients?***	
o *Yes*	
o *No*	

### Literature review

An extensive non-systematic literature review was independently assessed by two authors (AP, PF) on two electronic databases (PubMed, Scopus). Controversies were solved by discussion with other authors.

### Data analyses and statistical methods

Data were extrapolated from the SurveyMonkey® online software package and stored as an excel file (Microsoft Corp, Redmond, WA®). The results are presented as numbers and percentages. Descriptive statistics was computed for each question. All statistical analyses were assessed by using the SPSS statistical software® (Version 23).

## Results

Overall, 129 chiefs of 129 Italian neurosurgical departments were preliminary included, of whom only 47 (36%) answered to the questions raised by this Survey.

### Epidemiology of anticoagulation in neurosurgery

**Question 1:** *In the last year, indicate the approximate percentage of patients admitted in your department with “acute” neurosurgical indication -*e.g. *acute/chronic epidural-subdural hematomas, ICH, Subarachnoid Hemorrhage (SAH), Traumatic Subarachnoid Hemorrhage (tSAH)- in anticoagulant therapy.*

The majority of participants (60%) affirmed that 10–25% of patients with acute surgical indication were on anticoagulant therapy, while 26% of participants affirmed that less than 10% of patients were on anticoagulant therapy (Supplementary Fig. 1).

### Anticoagulated patients in emergency neurosurgery

**Question 2:** *In the clinical scenario of the above mentioned acute neurosurgical pathologies, do you apply a “forced” emergency reversal of anticoagulant agents?*

The option “yes, also if a conservative treatment is planned” was answered by 51% of the responders, while 49% of the participants choose the option “only if a surgical treatment is planned”.

### VKAs reversal therapy

**Question 3:** *In the eventuality of the clinical scenario of the above mentioned “acute neurosurgical pathologies”, how do you reverse VKA patients?*

**Fig. 1:**
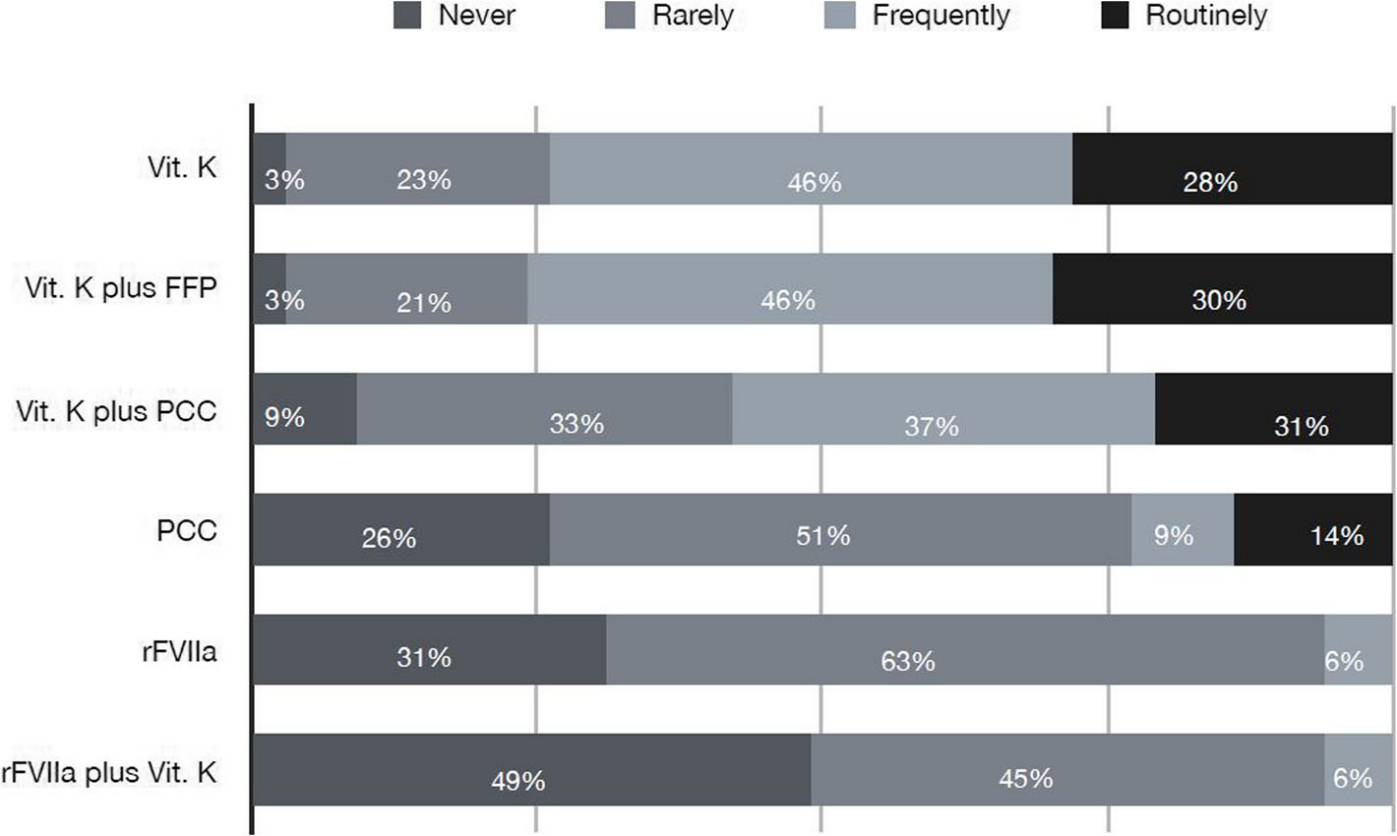
*QUESTION 3: How do you reverse VKA patients in “acute neurosurgical pathologies”? For every listed reversal agent, participants had to state if they use it routinely, frequently, rarely or never*

Most of the responders stated that they frequently or routinely use Vitamin K (45 and 29% respectively) or Vitamin K and FFP (46 and 30% respectively). The association vitamin K and PCC is frequently/routinely used in 37–21% and, of note, rarely or never used in 33 and 9% respectively. Recombinant activated factor VII (rFVIIa) is rarely used (Fig. 1).

### DOACs reversal therapy

**Question 4:** *In the eventuality of the clinical scenario of the above mentioned (“acute neurosurgical pathologies”), how do you reverse DOAC patients?*

**Fig. 2:**
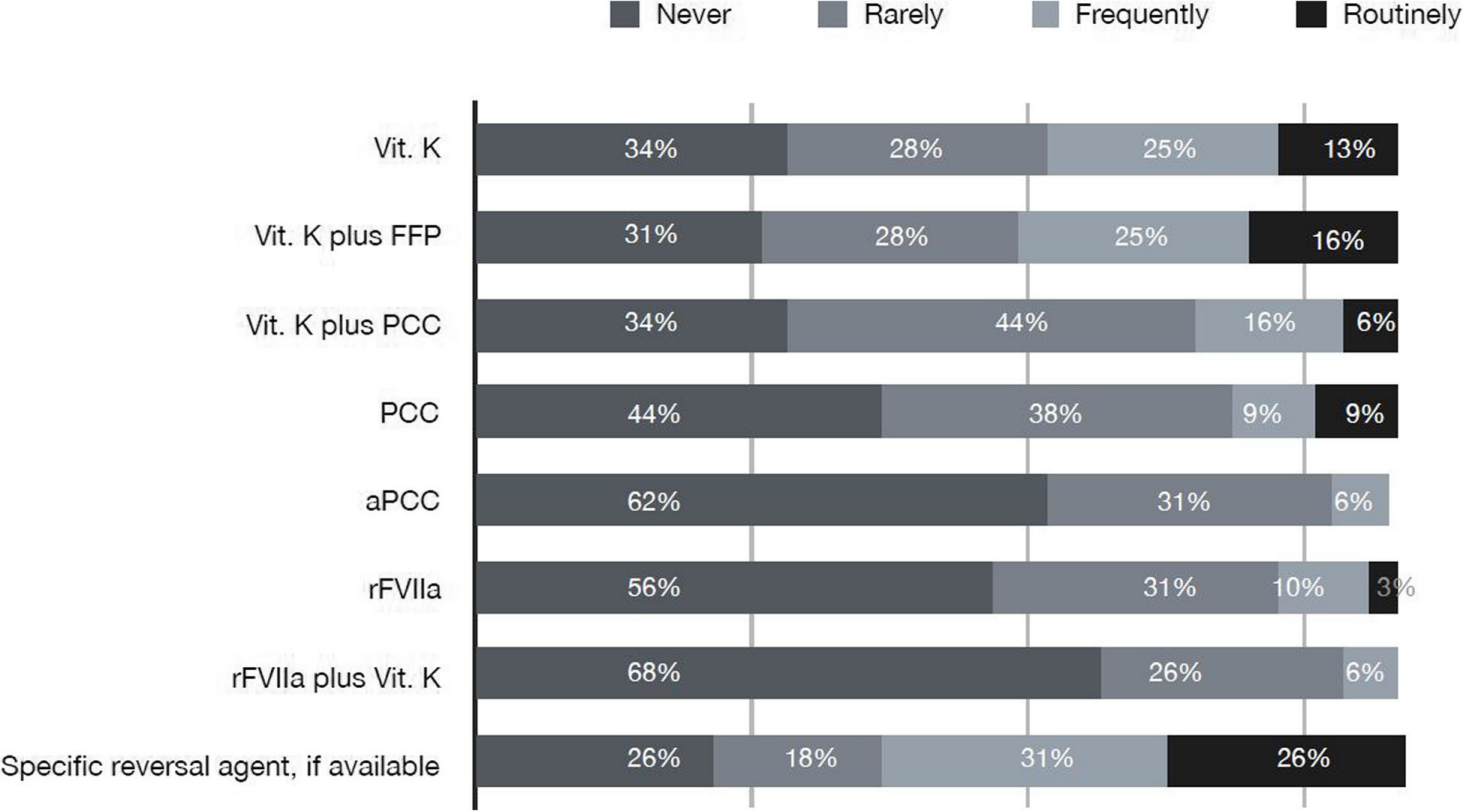
*QUESTION 4*: *How do you reverse DOAC patients in “acute neurosurgical pathologies”?* For every listed reversal agent, participants had to state if they use it routinely, frequently, rarely or never

A specific reversal agent (if available) is used frequently or routinely by 56% of the responder; 37,5 and 42% of responders stated that they used frequently or routinely Vitamin K and Vitamin K and FFP respectively. PCC and aPCC are used frequently or routinely only by 19 and 10% of responders respectively (the vast majority of the responders stated that they rarely or never use these agents, Fig. 2).

### Coagulation tests in DOACs

**Question 5:** *How do you assess anticoagulant effects in patients on DOACSs with acute neurosurgical pathologies? (multiple answers possible).*

**Fig. 3:**
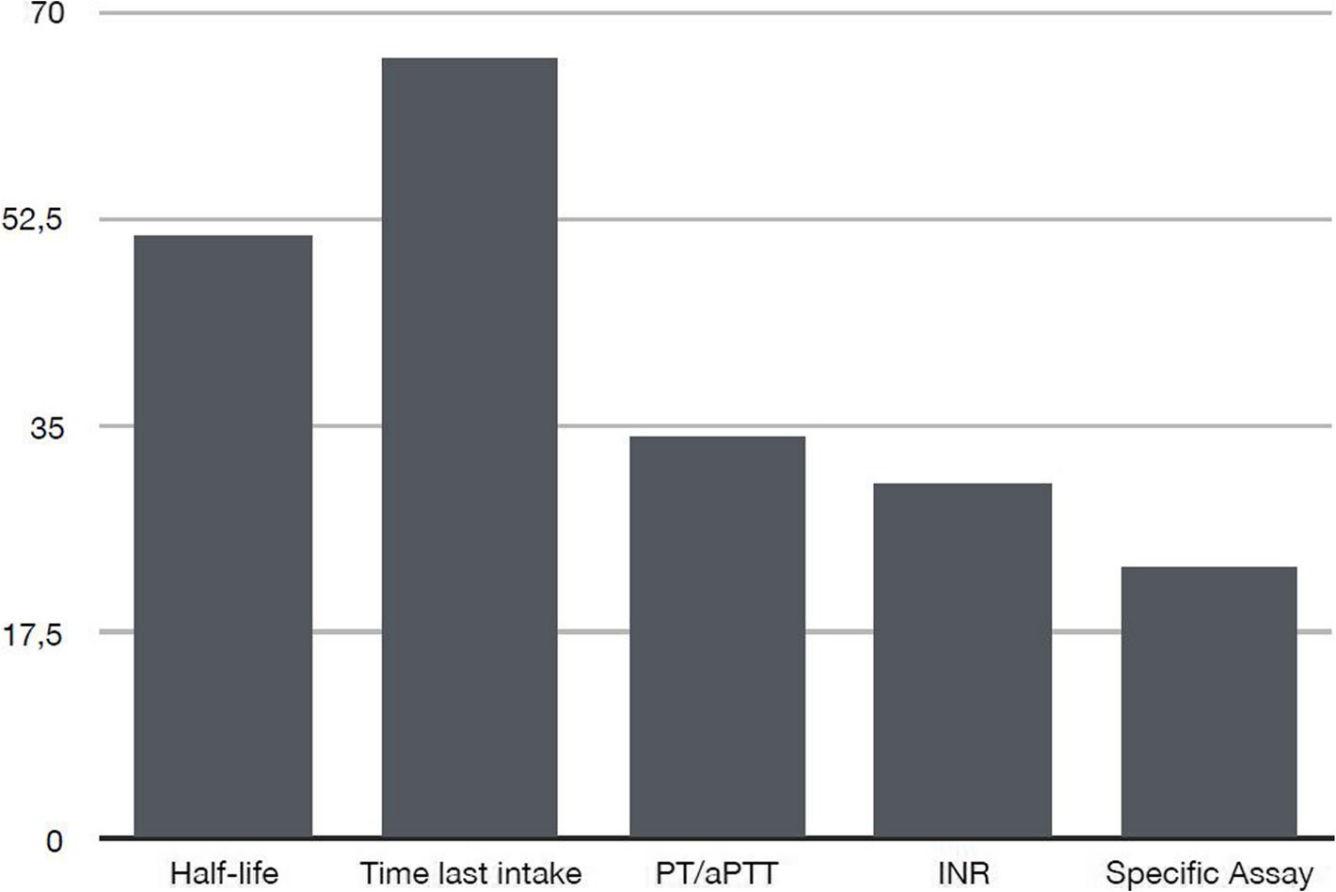
*QUESTION 6: How do you assess anticoagulant effects in patients on DOACSs with acute neurosurgical pathologies? Possible answers: drug’s half-life, time from the last intake of the drug, PT/aPTT, INR, specific assay. Multiple answers*

Most of the participants (66%) indicated that they try to determinate when the last dose was taken and 51% that they evaluate the half-life of the drug. The answer prothrombin time (PT) and activated partial thromboplastin time (aPTT) was chosen by 34% of the participants. Only 23% of responders used to assess the anticoagulant effect with a specific assay (Fig. 3).

### Anti-thrombotic therapy resumption

**Questions 6, 7 and 8**: *In your opinion, what is the optimal timing for anti-thrombotic therapy resumption in patients at high thrombotic risk (*e.g. *valvular atrial fibrillation, ventricular devices), moderate thrombotic risk (*e.g. *non-valvular atrial fibrillation) and low-thrombotic risk (*e.g. *previous history of deep venous thrombosis)?*

In patients at high thrombotic risk, 34% of participants re-initiateanti-thrombotic therapy less than 5 days after an intracranial bleeding or after elective surgery, 47% after 6 to 10 days and 19% after more than 10 days. In patients at moderate thrombotic risk, 15% of responders choose the answer “less than 5 days”, 38% the answer “between 6 to 10 days” and 47% the answer “more than 10 days”. Finally, in patients at low-thrombotic risk the majority (62%) of participants re-initiateanti-thrombotic therapy after more than 10 days, while 17% of participants after less than 5 days and 21% between 6 to 10 days (Supplementary Fig. 3).

### Perioperative management

**Question 9:** *What is the optimal timing for initiating venous thromboembolism prophylaxis after intracranial bleeding or after elective surgery?*

Most of the participants (64%) initiate chemoprophylaxis less than 2 days after intracranial bleeding or after elective surgery, 28% after 2 to 4 days, 2% after 4 to 7 days and 6% after more than 7 days (Supplementary Fig. 2).

**Question 10*:****Do you usually ask for a cardiological evaluation for the perioperative management of anticoagulated patients?*

Neurosurgical patients usually undergo cardiological evaluation (87%).

## Discussion

This national survey provides pivotal information regarding the management of anticoagulation among distinct neurosurgical departments in Italy. The main findings of our survey can be summarized as follow: 1) patients who undergo acute neurosurgical procedures are on anticoagulation in 10–25% of cases; 2) both in planned and unplanned neurosurgical procedures reversal of anticoagulation is frequently adopted; 3) Vitamin K or Vitamin K and FFP are mostly used for reversal of VKAs, while rFVIIa is rarely adopted; 4) when available, a specific reversal agent is preferred, although most of participants frequently or routinely use Vitamin K or Vitamin K and FFP; 5) Pharmacokinetic of anticoagulants is frequently investigated, general coagulation test are commonly used, while specific blood dosages of DOACs are adopted in about 20% of cases; 6) Prophylactic antithrombotic agents are most commonly prescribed between 6 to 10 days after intracranial bleeding or elective surgery; 7), 8), and 9) In patients with high thrombotic risk, antithrombotic agents in therapeutic dosage are commonly prescribed between 6 to 10 days after intracranial bleeding or elective surgery. In case of moderate risk, antithrombotic agents in therapeutic dosage are most frequently prescribed after 10 days from the surgical event, while for low-thrombotic risk antithrombotic drugs are reinitiated 10 days thereafter; 10) A perioperative cardiological evaluation is considered part of daily clinical practice in 87% of cases.

### 1) Epidemiology of anticoagulation in neurosurgery

Even though the exact prevalence of antithrombotic therapy in neurosurgical patients is unknown, data from recent studies suggested that many neurosurgical patients are on anticoagulation at the time of both elective and emergent presentation. Importantly, an increasing number of patients are treated with DOACs. In patients with atrial fibrillation (AF) who are eligible, DOACs are recommended over vitamin K antagonist (VKA) [8] . One of the major concerns related to DOACs is the lack of antidotes compared to VKAs; nevertheless in 2015 the FDA approved Idaracizumab for the reversal of dabigatran-induced coagulopathy in patients with life-threatening or uncontrolled bleeding and in patients requiring emergent/urgent procedures. Very recently, Andexanet Alfa has been approved by FDA for reversal of anti-activated factor X (FXa) drugs.

#### *Spontaneous ICH in Anticoagulated patients*

In a recent large registry-based retrospective cohort study including patients with intracerebral hemorrhage, 10.6% of patients received warfarin and 3.5% received DOACs prior to ICH [9]. Similarly, in a retrospective cohort study at 19 German tertiary care centers including 10.208 consecutive patients with ICH authors found a period prevalence rate of 13.0% of anticoagulant-related ICH [10]. Warfarin use increases the risk of primary ICH by 2–5% [11]). Of all spontaneous ICH cases 15–20% are anticoagulated by warfarin. Risk of ICH for patient taking Warfarin is approximately 1% per year [12–15] and as high as 1.9% in a cohort study [16]. Anticoagulant-associated intracranial hemorrhage is predictive of larger hematoma volumes, higher rates of hematoma expansion, and worse clinical outcomes as compared with spontaneous intracerebral hemorrhage in nonanticoagulated patients [2, 17].

DOACs seems to carry half the risk for ICH compared to vitamin K antagonists [18–21]. Moreover, a recent multicenter cross-sectional observational study suggests that DOAC-related ICHs appear to have more favorable neuroimaging (lower baseline median ICH volume) and clinical profiles (lower median NIH Stroke Scale scores -NIHSS- at admission and higher median admission Glasgow Coma Scale scores) on hospital admission compared to VKA-related ICH [22]. DOAC-related ICH was also associated with lower odds of 3-month disability and greater likelihood of 3-month functional improvement. In the context of ICH expansion, the literature provides conflicting results; some studies recorded lower rate of hematoma expansion in DOACs related versus VKA-related ICH [22–24] whereas others showed similar radiological evolution [25, 26].

#### *TBI in Anticoagulated patients*

Data regarding TBI in anticoagulated patients are more difficult to obtain. In a retrospective cohort study including 384 patients 55 years of age or older with closed head injuries over an 8-year period (April 1993–2001) 9% of patients were receiving warfarin before their trauma [27]. Gaist et al. extrapolate from the Danish National Patient Register 10,010 patients with a first-ever subdural hematoma discharge diagnosis from 2000 to 2015; 14.3% of patients were taking VKA and 1.0% were taking DOACSs [28]. Authors noted increasing incidence of subdural hematoma during the study period, especially among older patients; this fact seems to be temporally related to an increase in the use of antithrombotic drugs. In a more recent retrospective study conducted in Frankfurt am Main, Germany, authors analyzed 116 consecutive patients with acute SDH treated from January 2007 to June 2016 and they found that 54.1% of patients were on vitamin K antagonists and 8.1% were on DOACs at the time of TBI [29]. Anticoagulated patients who experienced a traumatic brain injury have higher risk of intracranial bleeding [29], a higher risk of hematoma expansion or delayed hematoma formation and increased morbidity and mortality [30–33]. Patients that have sustained a head injury while on Warfarin had a 2 to 6 times higher mortality than non-anticoagulated patients [34, 35]; outcome severity seems to be related to the degree of anticoagulation achieved by warfarin measured by international normalized ratio (INR) [36].

DOACSs patients seems to have the same initial hematoma volume, midline shift and functional outcome when compared to VKA related TBI [29].

### 2) Anticoagulated patients in emergency neurosurgery

#### *Management of VKA patients*

As stated by the Neurocritical Care Society [37] prompt reversal therapy is mandatory for patients with Vitamin K antagonists-related intracranial hemorrhage (both traumatic and spontaneous), irrespective of hematoma size, location or the indication for anticoagulation. Only for patients with negative neurological examination and very mild INR elevation (< 2) a conservative management may be evaluated, and risk and benefit of Vitamin K reversal must be balanced [17, 38–43]..

##### VKA associated spontaneous Intracerebral hemorrhage (sICH)

In the European Stroke Organization (ESO) guidelines for the management of spontaneous intracerebral hemorrhage [44] authors stated that, due to the absence of RCTs, they cannot make strong recommendation about reversal therapy for patients with spontaneous ICH taking anticoagulant drugs. However, they suggest stopping anticoagulant medication if INR was elevated, to give intravenous vitamin K and to add fresh-frozen plasma (FFP) or prothrombic complex concentrates (PCC) to prevent hematoma expansion. Rapid reversal of VKA and normalization of INR, alongside with specific systolic blood pressure management, leads to lower rates of hematoma enlargement and to reduced mortality [45–47]. In a large retrospective cohort study Kuramatsu et al. [10] noted that INR reversal to values below 1.3 achieved within 4 h after admission was associated with fewest rates of hematoma enlargement.

##### VKA associated TBI

There is little consensus regarding management of patients with TBI taking anticoagulants. Discontinuation of anticoagulant therapy is recommended but it is not clear if rapid INR reversal is associated with a better outcome. Ivascu et al. [38] reported 11% rates of hematoma expansion in patients taking Warfarin and with an initial positive CT scan rapidly treated with FFP and vitamin K versus 40% of progression rate in patients without FFP infusion. Mortality also decreased significantly in reversed patients (10% compared with the 50% rate noted in controls). Hans Andrews et al. [48] retrospectively review 100 patients with traumatic intracranial hemorrhage taking prehospital anticoagulant medication; they stated that reversal of elevated INR levels in less than 10 h significantly decreases the likelihood of intracranial hemorrhage expansion. In a recent published article Watson et al. [49] proposed interesting guidelines for the management of TBI patients on antithrombotic therapy initially considered non-operative. Reversal therapy was guided by INR values, Platelet Function Assays, CT scan and neurological examination. This comprehensive evaluation enables a simple and individualized approach to the patient assuming anticoagulant or antiplatelet drugs; this could lead to a better and faster management for this kind of patients.

#### *Management of DOACs patients*

Similarly to patients with VKA associated intracranial hemorrhage, discontinuation of the drug, blood pressure management and correction of the coagulation status is needed to limit hematoma enlargement in TBI patients under DOACs [50]. If a DOAC-treated patients require an urgent surgical procedure it is important to determine which DOACs is taken and when the last dose was taken; if the last DOAC dose was taken at least 24 h before urgent surgery, it is likely that more than 80%of the therapeutic effect will be gone in the presence of normal kidney function (because of the short half-lives of this medications) [51]. Measuring the anticoagulant effect of DOACs with specific coagulation tests (see next chapter) may be helpful in these situations. If the procedure cannot be delayed and the patient’s coagulation status indicates a high risk of bleeding, DOAC reversal should be considered before the procedure [52].

### 3) VKAs reversal therapy

VKAs: Vitamin K antagonists exert their effect by antagonizing vitamin K in the synthesis of clotting factors II, VII, IX and X. Vitamin K normalizes the INR by providing the necessary substrate to synthesize these coagulation factors [53, 54]; nevertheless reduction of INR to values less than 1.4 may take up to 24 h [55, 56] because Vitamin K effect is delayed by the requirement of de novo synthesis of clotting factors by the liver [57]. For this reason in the acute setting vitamin K monotherapy is not adequate and should be supplemented with vitamin K-dependent coagulation factors [53, 55, 56, 58–63].

#### FFP, PCC and rFVIIa

FFP is plasma isolated from a unit of whole blood and contains all clotting factors. PCC contains a mixture of vitamin K-dependent clotting factors (II, IX and X in the three factor PCC -3PCC-, II, VII, IX and X in the four factor PCC -4PCC-). Traditionally, FFP in combination with vitamin K was considered as first choice treatment for VKA reversal. Recently, the INCH trial stated that patient with VKA-ICH were more likely to normalize INR within 3 h after administration of 4PCC in comparison to administration of FFP. Patients treated with PCC had significantly less hematoma and lower mortality while no effect was seen on the mRS at 90 days between the two groups [43]. FFP requires longer time for INR normalization in comparison to PCC because of the lower concentration of clotting factors and the time needed to safely transfuse larger volumes of FFP [64, 65];most of the patients require more than 1 unit of FFP to normalize INR and this could lead to excessive amounts of volume given to the patient, which can cause volume overload and result in pulmonary edema especially in patient with impaired cardiac function [46]. Other drawbacks of using FFP are the potential for transfusion-related reaction such as acute lung injury; moreover, FFP need to be cross-matched before used [66]. On the other hands FFP has lower cost and widespread availability compared to PCC [44]. Both FFP and PCC carries a small risk of viral infection transmission [57] and similar rates of thrombotic complications (3–8%) [67, 68]. Four-factor PCC seems to be superior to three-factor PCC for INR reversal, but only two retrospective studies have directly compared 3PCC with 4 PCC and there are no data from prospective randomized trials [69, 70]. Of note pharmacologic effects of PCC begin to wane after approximately 12–24 h [60]. Despite little data are available, PCC multiple dose is not recommended [71]; in case of inadequate INR-value after a full dose of PCC, the administration of FFP may be considered [44].

rFVIIa is a vitamin K-dependent glycoprotein structurally similar to human-derived factor VII that enhances thrombin generation [57]. It is fast in reduce INR but has been associated with higher thrombosis rate (12,8–24%) [72–75]. Another drawback is the high cost of treatment [76, 77].

### 4) DOACs reversal therapy

#### *Non-specific reversal agents*

Prothrombic complex concentrates (PCCs) are the preferred non-specific hemostatic agent for DOACS reversal [8]. Animal experiments and studies in healthy volunteers have indicated the efficacy of PCCs and aPCC for the normalization of coagulation parameters under DOACs treatment [50] but it is still unclear if normalization of coagulation parameter translates to improved outcomes in patients with acute bleeding. Indeed, in a recent German retrospective multicenter cohort study authors demonstrated that hemostatic treatment with PCC was not associated with a reduced risk of hematoma enlargement, mortality, or unfavorable functional outcome either in overall DOACS-related ICH or specifically in factor Xa inhibitor–related ICH [47]. Of note in this study patients received a PCC weight-adjusted dosage ≥25 IU/kg bodyweight while updated guidelines now refers to dosages ≥50 IU/kg bodyweight [37].

#### Specific reversal agents

A specific reversal agent is available for dabigatran (idarucizumab, a humanized antibody fragment that specifically binds dabigatran) [78]. Specific agents for FXa inhibitors are andexanet alfa (a recombinant human FXa analogue that competes with FXa to bind FXa inhibitors) [79] and ciraparantag [80]. Importantly, even after direct reversal therapy significant DOACs concentrations may reappear in some patients and contribute to recurrent or continued bleeding (particularly after andexanet alpha, less frequently after Idarucizumab administration), underlining the necessity for continued clinical and laboratory monitoring [50].

Idaracizumab: Idaracizumab was approved in 2015 by FDA for the reversal of dabigatran-induced coagulopathy. In a phase 3, global, prospective, cohort study [78] Idaracizumab completely reversed the anticoagulant activity of dabigatran within minutes in almost all patients. As stated before continued clinical and laboratory monitoring is recommended, since a dose of Idarucizumab may not completely neutralize an exceptional high level of dabigatran [50].

Andexanet Alfa: this reversal agent has been approved recently (May 2018) by FDA on the basis of two Phase 3 ANNEXA studies (ANNEXA-R and ANNEXA-A) that demonstrated that Andexanet Alfa rapidly and significantly reversed anti-Factor Xa in healthy volunteers. The ANNEXA-4 study [81] confirmed the hemostatic efficacy of andexanet in patients with acute major bleeding associated with the use of a factor Xa inhibitor.

### Conclusions/recommendations

Patients with Vitamin K antagonists or DOACs-related intracranial hemorrhage should be promptly reverted (except in very selected cases). A target value of < 1.5 for INR is recommended [63]. Vitamin K is a mainstay for reversal therapy of VKA and should be administered immediately to patients with VKA-associated intracranial hemorrhage; nevertheless, it must be supplemented with FFP or PCC. PCC (preferably 4 factors PCC) is recommended [63]; PCC dosing should be weight-based and vary according to the INR values^38^. FFP may be considered when PCC is not available, in those with a history of allergy or adverse reaction to PCC or its components or in patients that have already received a full dose of PCC but do not have adequate INR correction. rFVIIa use for VKA reversal in patients with intracranial hemorrhage is not recommended except in selected circumstances.

In patients on DOACs therapy, measuring the anticoagulant effect of DOACs may be helpful to guide emergency care. In dabigatran-treated patients, Idarucizumab should be considered as the first-line option, while in direct factor Xa inhibitor-treated patients Andexanet Alfa is now available. If specific reversal agents are not available, administration of PCC should be considered, or alternatively, administration of aPCC or rFVIIa may be considered. FFP should not be used as a reversal agent unless no coagulation factor concentrate is available. Haemodialysis may be considered in dabigatran-treated patients, particularly in case of impaired renal function, if Idarucizumab is not available.

### 5) Coagulation tests in DOACs

Routine coagulation tests do not provide an accurate assessment of DOACs anticoagulant effects [63]. Prolonged activated partial thromboplastin time (aPTT) indicates an anticoagulant effect of dabigatran, and a prolonged prothrombin time (PT) indicates anticoagulant effect of the FXa inhibitors. However, the clinical utility of these common tests is limited due to the fact that a normal aPTT or PT does not exclude clinically relevant plasma levels of dabigatran and FXa inhibitors [82, 83]. More accurate tests to measure plasma dabigatran include the diluted thrombin time assay, the ecarin clotting time assay, or the ecarin chromogenic assay [84, 85], while measurement of the anticoagulant activity of direct factor Xa inhibitors can be determined with calibrated chromogenic anti-FXa assays [86, 87]. Unfortunately, these more sensitive tests are hampered by lack of standardization and limited availability [88, 89].

### 6), 7), 8) anti-thrombotic therapy resumption

Resumption of antithrombosis postoperatively requires balancing the risks of rebleeding with the risks of thromboembolic complications if antithrombotic are not resumed. Postoperative hemorrhagic risk can be categorized as low (2- day risk of major bleeding 0–2%) or high (2-day risk of major bleeding 2–4%) and neurosurgical procedures are considered to have a high risk of bleeding. Thromboembolic risk can be estimation by calculate the CHA2DS2-VASc score (low risk if or 1), with an additional increase in risk in case of recent stroke or recent pulmonary embolism [8].

Optimal timing for the resumption of VKA after elective surgery is not precisely provided by the literature. In the “2017 Expert Consensus Decision Pathway for Periprocedural Management of Anticoagulation in Patients With Nonvalvular Atrial Fibrillation” authors stated that if hemostasis is achieved, VKA can be restarted in the first 24 h after the procedure in most situations, also because VKA anticoagulant effect typically begins 24 to 72 h after initiation of therapy and the full therapeutic effect occurs 5 to 7 days after re-initiation [90]. Nevertheless in the setting of a procedure at high risk for bleeding delayed re-initiation of anticoagulation may be considered [90]. In a review that considered anticoagulated patients undergoing neurosurgical procedures, authors developed recommendations based on thromboembolic risk status (high risk, e.g. onset of deep venous thrombosis or pulmonary embolism within the three months before surgery, moderate risk, e.g. patients with mechanical valves, and low risk). Authors recommended that high-risk patients should receive a vena cava filter and subcutaneous heparin, and oral anticoagulation should be restarted on postoperative days 3–5. Moderate-risk patients should receive subcutaneous heparin postoperatively, and oral anticoagulation should be restarted on postoperative days 5–7. Low-risk patients should receive subcutaneous heparin postoperatively, and oral anticoagulation should be restarted on postoperative days 7–14 [91]. The European Heart Rhythm Association Practical Guide on DOACs management suggests, after planned surgery, initiation of post-operative thromboprophylaxis 6–8 h after surgery and restarting the DOACSs 48–72 h postoperatively [50]. In a German survey regarding the perioperative management of anticoagulated patients most of the responders restarted VKA or DOACs 8–14 days after surgery [92].

Regarding TBI, literature demonstrate that resumption of anticoagulant treatment was associated with a net benefit for patients in terms of a reduction in risk of stroke [93, 94]; patients with a history of prior antithrombotic therapy experience thromboembolic complications earlier after TBI, with a peakin the first 10 days post-trauma [95]. Nevertheless, the optimal time of anticoagulant therapy resumption is not clear. In a recent literature review, authors stated that after TBI it might be reasonable to resume anticoagulation (warfarin/DOACs) from day 3 after the hemorrhage in patients at high risk of thromboembolism with bridge therapy, and from day 7 in patients at low risk [96]; this approach may carry an acceptably low risk of hemorrhagic complications (of note hemorrhagic complications may be lower with the introduction of NOACs than with VKAs). In “The 2018 European Heart Rhythm Association Practical Guide on the use of non-vitamin K antagonist oral anticoagulants in patients with atrial fibrillation authors stated that after evacuation of traumatic epidural or subdural hematoma anticoagulation could be safely reinitiated about 4 weeks after surgery [50].

A very frequent dilemma for neurosurgeon is the time of anticoagulant resumption in patients under chronic anticoagulant therapy operated for chronic subdural hematoma (cSDH). It seems that anticoagulant could be associated with higher postoperative recurrence rate [97, 98], but risk of rebleeding has to be balanced with the risk of thromboembolic complications. In a retrospective analysis Guha et all evaluated patients under either antiplatelets or anticoagulants drugs that underwent surgical evacuation of cSDH; they found that patients with a history of preoperative antithrombotic therapy experienced thromboembolic complications significantly earlier than patients without antithrombotic drugs. They stated that resuming chronic anticoagulant therapy early following the surgical evacuation of cSDH (3 days postoperatively) may be safe [97], but the literature is not conclusive about timing of anticoagulant resumption, ranging from 3 to 5 days to 7 days to several weeks.

Another major clinical dilemma is if and when resume antithrombotic therapy after spontaneous ICH; anticoagulated patients that survives after sICH are at risk of ICH recurrence, but also of ischemic stroke. The risk of ischemic stroke in people with AF is should be estimated using the CHA2DS2VASC score and it seems reasonable to use this score in populations of ICH survivors [99], but the risk of ICH recurrence is more difficult to estimate. In a recent systematic review the rate of recurrence was between 1.8 and 7.4% [100], and it was higher after “lobar” ICH in comparison with “deep” ICH [100]. The difference is related to the different underlying small vessel disease, being lobar ICH more frequently associated with cerebral amyloid angiopathy (CAA) [101]. Beyond the ICH location defined by the CT scan, MRI can be used to diagnose CAA with high specificity in ICH patients with the identification of biomarkers of small vessel disease, including distribution of cerebral microbleeds (CMBs) [102]. Given the high risk of ICH recurrence, some authors recommend that a patient with AF and a probable CAA should not be anticoagulated [50, 103]; nevertheless a recent meta-analysis indicate decreased mortality and favorable functional outcome after resumption of oral anticoagulation after intracerebral bleeding [104]. Given the fact that DOACs have a ~ 50% lower ICH risk than VKA, anticoagulation with DOACSs should be preferred in most ICH survivors, except where warfarin is indicated (e.g. in those with metallic mechanical heart valves) [8]. An important recent development approved by FDA is the left atrial appendage occlusion (LAAO). As LAAO seems to have similar efficacy to oral anticoagulation in patients with AF, it could be a potential substitute for long-term anticoagulation in AF patients post-ICH and an option for ICH survivors at high risk of recurrent ICH (e.g. those with probable cerebral amyloid angiopathy). Nevertheless randomized trials are needed to definitively determine the safety and efficacy of LAAO in ICH survivors [50]. Regarding the timing of anticoagulant resumption, there are no reliable randomized trial data to guide clinicians; resumption should be delayed beyond the acute phase (~ 48 h) and probably for at least 4–8 weeks [8, 50].

### 9), 10) perioperative management

Perioperative management of anticoagulation therapy is a delicate balance between the risk of thrombotic complications versus bleeding risk; in this view, a multidisciplinary team consensus is advocated [50, 63, 105]. We already discussed about the issue of anticoagulant therapy resumption, therefore in this chapter we’ll briefly review the management of anticoagulant suspension before planned surgery.

### Timing of anticoagulant suspension before planned surgery and bridging

Neurosurgical procedures are considered “high hemorrhagic risk surgery”; this means that the risk of bleeding both in the intra-operative and immediate post-operative period is substantial and that anticoagulant interruption is mandatory to minimize the hemorrhagic risk [8, 50].

#### VKA

Discontinuation of warfarin is usually instituted 5 days before an elective surgical intervention, with INR checked before surgery. Surgery can be regularly planned if the INR is ≤1.4–1.5 the day before surgery or the same day of surgery [106].

#### DOACS

High hemorrhagic risk surgery should be performed when less than 10% of the residual anticoagulant effect exists [51]. It is therefore recommended to take the last DOACS dose 48 h or longer before surgery [50]. In patients in whom DOACS clearance is unclear (e.g. patient with impaired renal function, older patients or patients assuming medication with potential DOACs interaction) measurement of DOACs plasma levels with specific coagulation tests may be considered [50].

### Venous thromboembolism prophylaxis

Neurosurgical patients are considered at moderate-high risk for venous thromboembolism (VTE), both after acute conditions such as TBI or hemorrhagic stroke [57] and following elective neurosurgical procedures [107]. After TBI, the most appropriate approach for VTE prophylaxis is the use of mechanical prophylaxis, in the form of graduated compression stockings (CS) and intermittent pneumatic compression (IPC) devices, together with low molecular-weight heparin (LMWH) or low-dose unfractionated heparin [96]. After TBI or hemorrhagic stroke, early administration of low-dose subcutaneous heparin decrease the risk of thromboembolism without increasing the risk of rebleeding [108, 109]; chemical prophylaxis may be initiated 24 h after CT documenting stability of intracranial blood and the absence of new hemorrhage [57, 108]. Chemoprophylaxis along with mechanical prophylaxis is suggested after elective neurosurgical procedures [57]; in a recent meta-analysis, Khan et al. concluded that chemoprophylaxis is beneficial in preventing VTE with no significant increase in either major or minor bleeding complications [107]. Initiating chemoprophylaxis 24 h following a cranial or spinal surgical procedure has been shown to be safe [57].

### Bridging periods

The BRIDGE trial (a trial performed in patients on warfarin candidate to an invasive procedure, patients with mechanical valves were excluded), demonstrated that perioperative anticoagulation with heparin/LMWH didn’t reduce cardiovascular events when compared to no bridging [110]. Notably, a significantly higher risk of major bleeding was observed; the results of this trial discourage pre-operative bridging in patients without high risk of thromboembolism or without a mechanical valve [8]. Pre-operative bridging (LMWH at therapeutic doses starting 3 days before the procedure) can be considered in patients receiving VKA who are particularly high risk of TE (e.g., recent stroke, mechanical heart valve) [106].

## Conclusions

Anticoagulation therapy is gaining attention in neurosurgical practice. Our survey revealed that there is lack of uniformity concerning the management of anticoagulation in Italy, mostly regarding the indication and strategies for the reversal of anticoagulants. This may be due to lack of national and international guidelines for the care of anticoagulation in neurosurgical patients. Meanwhile, establishment of a multidisciplinary hospital-wide policy concerning the management of anticoagulated neurosurgical patients is strongly recommended.

## Supplementary Information

**Additional file 1: Supplementary Fig. 1.** QUESTION 1: In the last year, indicate the approximate percentage of patients admitted in your department with “acute” neurosurgical indication. Possible answers: less than 10%, between 10 and 25%, between 25 and 50%, between 50 and 75%, more than 75%.

**Additional file 2: Supplementary Fig. 2.** QUESTION 6: What is the optimal timing for initiating venous thromboembolism chemoprophylaxis after intracranial bleeding or after elective surgery? Possible answers: less than 2 days, between 2 and 4 days, between 4 and 7 days and more than 7 days

**Additional file 3: Supplementary Fig. 3.** QUESTIONS 8, 9 and 10: In your opinion, what is the optimal timing for anti-thrombotic therapy resumption in patients at high thrombotic risk (e.g. valvular atrial fibrillation, ventricular devices), moderate thrombotic risk (e.g. non-valvular atrial fibrillation) and low-thrombotic risk (e.g. previous history of deep venous thrombosis)?

**Additional file 4.**

## Data Availability

Not Appliable, no potentially identifying information are included in the study.

## Abbreviations

AF: atrial fibrillation
aPCC: activated prothrombic complex concentrates
aPTT: activated partial thromboplastin time
CAA: cerebral amyloid angiopathy
CMBs: cerebral microbleeds
CS: compression stockings
cSDH: chronic subdural hematoma
CT: computed tomography
DOACs: direct oral anticoagulants
FXa: activated factor X
FDA: food and drug administration
FFP: fresh-frozen plasma
ICH: intracerebral hemorrhage
INR: international normalized ratio
LAAO: left atrial appendage occlusion
LMWH: low molecular-weight heparin
NIHSS: NIH Stroke Scale
PCC: prothrombic complex concentrates
PT: prothrombin time
rFVIIa: recombinant activated factor VII
SAH: subarachnoid hemorrhage
sICH: spontaneous intracerebral hemorrhage
TBI: traumatic brain injury
VKA: vitamin k antagonist
VTE: venous thromboembolism

## References

[1] Gillespie L, Gillespie W, Robertson W, Lamb S, Cumming R, Rowe B. Interventions for preventing falls in elderly people. ACP J Club. 2002. 10.1002/14651858.CD000340 14583918

[2] Flaherty ML, Kissela B, Woo D, Kleindorfer D, Alwell K, Sekar P, et al. The increasing incidence of anticoagulant-associated intracerebral hemorrhage. Neurology. 2007. 10.1212/01.wnl.0000250340.05202.8b 17210891

[3] Veltkamp R, Rizos T, Horstmann S. Intracerebral bleeding in patients on antithrombotic agents. Semin Thromb Hemost. 2013. 10.1055/s-0033-1357506 24114010

[4] Croci DM, Kamenova M, Guzman R, Mariani L, Soleman J (2017). Novel Oral anticoagulants in patients undergoing cranial surgery. World Neurosurg..

[5] Faraoni D, Comes RF, Geerts W, Wiles MD (2018). European guidelines on perioperative venous thromboembolism prophylaxis. Eur J Anaesthesiol.

[6] Albaladejo P, Bonhomme F, Blais N, Collet JP, Faraoni D, Fontana P (2017). Management of direct oral anticoagulants in patients undergoing elective surgeries and invasive procedures: updated guidelines from the French working group on perioperative hemostasis (GIHP) – September 2015. Anaesth Crit Care Pain Med.

[7] Fiaschi P, Iaccarino C, Stefini R, Prior E, Prior A, Zona G. Clinical practice for antiplatelet and anticoagulant therapy in neurosurgery: data from an Italian survey and summary of current recommendations – part I, antiplatelet therapy. Neurosurg Rev. 2020. 10.1007/s10143-019-01229-7 31953783

[8] Lip GYH, Banerjee A, Boriani G, Chiang CE, Fargo R, Freedman B (2018). Antithrombotic therapy for atrial fibrillation: CHEST guideline and expert panel report. Chest..

[9] Inohara T, Xian Y, Liang L, Matsouaka RA, Saver JL, Smith EE, et al. Association of Intracerebral Hemorrhage Among Patients Taking Non–Vitamin K Antagonist vs Vitamin K Antagonist Oral Anticoagulants With In-Hospital Mortality. JAMA. 2018. 10.1001/jama.2017.21917 PMC5839299 29372247

[10] Kuramatsu JB, Gerner ST, Schellinger PD, Glahn J, Endres M, Sobesky J, et al. Anticoagulant reversal, blood pressure levels, and anticoagulant resumption in patients with anticoagulation-related intracerebral hemorrhage. JAMA - J Am Med Assoc. 2015. 10.1001/jama.2015.0846 25710659

[11] Beshay JE, Morgan H, Madden C, Yu W, Sarode R. Emergency reversal of anticoagulation and antiplatelet therapies in neurosurgical patients. J Neurosurg. 2010. 10.3171/2009.7.JNS0982 19663548

[12] Connolly SJ, Ezekowitz MD, Yusuf S, Eikelboom J, Oldgren J, Parekh A, et al. Dabigatran versus warfarin in patients with atrial fibrillation. N Engl J Med. 2009.

[13] Patel MR, Mahaffey KW, Garg J, Pan G, Singer DE, Hacke W, et al. Rivaroxaban versus warfarin in nonvalvular atrial fibrillation. N Engl J Med. 2011. 10.1056/NEJMoa1009638 21830957

[14] Granger CB, Alexander JH, McMurray JJ V, Lopes RD, Hylek EM, Hanna M, et al. Apixaban versus warfarin in patients with atrial fibrillation. N Engl J Med. 2011.

[15] Giugliano RP, Ruff CT, Braunwald E, Murphy SA, Wiviott SD, Halperin JL, et al. Edoxaban versus Warfarin in Patients with Atrial Fibrillation. N Engl J Med. 2013. 10.1056/NEJMoa1310907 24251359

[16] Hylek EM, Evans-Molina C, Shea C, Henault LE, Regan S. major hemorrhage and tolerability of warfarin in the first year of therapy among elderly patients with atrial fibrillation. Circulation. 2007. 10.1161/CIRCULATIONAHA.106.653048 17515465

[17] Cucchiara B, Messe S, Sansing L, Kasner S, Lyden P. Hematoma growth in oral anticoagulant related intracerebral hemorrhage. Stroke. 2008. 10.1161/STROKEAHA.108.520668 18703803

[18] Caldeira D, Barra M, Pinto FJ, Ferreira JJ, Costa J. Intracranial hemorrhage risk with the new oral anticoagulants: a systematic review and meta-analysis. J Neurol. 2015. 10.1007/s00415-014-7462-0 25119841

[19] Hankey GJ. Intracranial hemorrhage and novel anticoagulants for atrial fibrillation: what have we learned? Current cardiology reports. 2014. 10.1007/s11886-014-0480-9 24643903

[20] Dogliotti A, Paolasso E, Giugliano RP. Novel oral anticoagulants in atrial fibrillation: a meta-analysis of large, randomized, controlled trials vs warfarin. Clin Cardiol. 2013. 10.1002/clc.22081 PMC6649520 23338902

[21] Ruff CT, Giugliano RP, Braunwald E, Hoffman EB, Deenadayalu N, Ezekowitz MD, et al. Comparison of the efficacy and safety of new oral anticoagulants with warfarin in patients with atrial fibrillation: a meta-analysis of randomised trials. Lancet. 2014. 10.1016/S0140-6736(13)62343-0 24315724

[22] Tsivgoulis G, Lioutas V-A, Varelas P, Katsanos AH, Goyal N, Mikulik R, et al. Direct oral anticoagulant– vs vitamin K antagonist–related nontraumatic intracerebral hemorrhage. Neurology. 2017. 10.1212/WNL.0000000000004362 28814457

[23] Kurogi R, Nishimura K, Nakai M, Kada A, Kamitani S, Nakagawara J, et al. Comparing intracerebral hemorrhages associated with direct oral anticoagulants or warfarin. Neurology. 2018. 10.1212/WNL.0000000000005207 PMC5880631 29490916

[24] Brelie Von der C, Doukas A, Naumann R, Dempfle A, Larsen N, Synowitz M, et al. Clinical and radiological course of intracerebral haemorrhage associated with the new non-vitamin K anticoagulants. Acta Neurochir (Wien). 2017;159:101–9. 10.1007/s00701-016-3026-7 27873051

[25] Purrucker JC, Haas K, Rizos T, Khan S, Wolf M, Hennerici MG, et al. Early Clinical and Radiological Course, Management, and Outcome of Intracerebral Hemorrhage Related to New Oral Anticoagulants. JAMA Neurol. 2015. 10.1001/jamaneurol.2015.3682 26660118

[26] Boulouis G, Morotti A, Pasi M, Goldstein JN, Gurol ME, Charidimou A. Outcome of intracerebral haemorrhage related to non-vitamin K antagonists oral anticoagulants versus vitamin K antagonists: a comprehensive systematic review and meta-analysis. J Neurol Neurosurg Psychiatry. 2018. 10.1136/jnnp-2017-316631 29030422

[27] Lavoie A, Ratte S, Clas D, Demers J, Moore L, Martin M, et al. Preinjury warfarin use among elderly patients with closed head injuries in a trauma center. J Trauma - Inj Infect Crit Care. 2004. 10.1097/01.ta.0000066183.02177.af 15187746

[28] Gaist D, Garcia Rodriguez LA, Hellfritzsch M, Poulsen FR, Halle B, Hallas J, et al. Association of antithrombotic drug use with subdural hematoma risk. JAMA - J Am Med Assoc. 2017. 10.1001/jama.2017.0639 28245322

[29] Won S-Y, Dubinski D, Bruder M, Cattani A, Seifert V, Konczalla J. Acute subdural hematoma in patients on oral anticoagulant therapy: management and outcome. Neurosurg Focus. 2017. 10.3171/2017.8.FOCUS17421 29088960

[30] Brewer ES, Reznikov B, Liberman RF, Baker R A, Rosenblatt MS, David C A, et al. Incidence and predictors of intracranial hemorrhage after minor head trauma in patients taking anticoagulant and antiplatelet medication. J Trauma. 2011. 10.1097/TA.0b013e3181e5e286 20693913

[31] Collins CE, Witkowski ER, Flahive JM, Anderson FA, Santry HP. Effect of preinjury warfarin use on outcomes after head trauma in Medicare beneficiaries. Am J Surg. 2014. 10.1016/j.amjsurg.2014.05.019 PMC4457283 25129426

[32] Dossett LA, Riesel JN, Griffin MR, Cotton BA. Prevalence and implications of preinjury warfarin use: an analysis of the National Trauma Databank. Arch Surg. 2011. 10.1001/archsurg.2010.313 21242422

[33] Prior A, D’Andrea A, Robba C, Fiaschi P. Letter to the editor regarding “first intracranial pressure monitoring or first operation: which one is better?”. World Neurosurg. 2020. 10.1016/j.wneu.2020.03.219 32298830

[34] Mountain D, Sistenich V, Jacobs IG (2010). Characteristics, management and outcomes of adults with major trauma taking pre-injury warfarin in a Western Australian population from 2000 to 2005: a population-based cohort study. Med J Aust.

[35] Mina AA, Knipfer JF, Park DY, Bair HA, Howells GA, Bendick PJ (2002). Intracranial complications of preinjury anticoagulation in trauma patients with head injury. J Trauma.

[36] Smith K, Weeks S. The impact of pre-injury anticoagulation therapy in the older adult patient experiencing a traumatic brain injury: a systematic review. JBI Libr Syst Rev. 2012. 10.11124/jbisrir-2012-429 27820526

[37] Frontera JA, Lewin JJ, Rabinstein AA, Aisiku IP, Alexandrov AW, Cook AM, et al. Guideline for reversal of Antithrombotics in intracranial hemorrhage: a statement for healthcare professionals from the Neurocritical care society and Society of Critical Care Medicine. Neurocrit Care. 2016. 10.1007/s12028-015-0222-x 26714677

[38] Ivascu FA, Howells GA, Junn FS, Bair HA, Bendick PJ, Janczyk RJ, et al. Rapid warfarin reversal in anticoagulated patients with traumatic intracranial hemorrhage reduces hemorrhage progression and mortality. J Trauma - Inj Infect Crit Care. 2005. 10.1097/01.ta.0000189067.16368.83 16385291

[39] Pieracci FM, Eachempati SR, Shou J, Hydo LJ, Barie PS. Degree of anticoagulation, but not warfarin use itself, predicts adverse outcomes after traumatic brain injury in elderly trauma patients. J Trauma. 2007. 10.1097/TA.0b013e31812e5216 18073596

[40] Franke CL, De Jonge J, Van Swieten JC, De Coul AAWO, Van Gijn J. Intracerebral hematomas during anticoagulant treatment. Stroke. 1990. 10.1161/01.str.21.5.726 2339452

[41] Rosand J, Eckman MH, Knudsen KA, Singer DE, Greenberg SM. The Effect of Warfarin and Intensity of Anticoagulation on Outcome of Intracerebral Hemorrhage. Arch Intern Med. 2004. 10.1001/archinte.164.8.880 15111374

[42] Flibotte JJ, Hagan N, O’Donnell J, Greenberg SM, Rosand J. Warfarin, hematoma expansion, and outcome of intracerebral hemorrhage. Neurology. 2004. 10.1212/01.wnl.0000138428.40673.83 15452298

[43] Steiner T, Poli S, Griebe M, Hüsing J, Hajda J, Freiberger A, et al. Fresh frozen plasma versus prothrombin complex concentrate in patients with intracranial haemorrhage related to vitamin K antagonists (INCH): a randomised trial. Lancet Neurol. 2016. 10.1016/S1474-4422(16)00110-1 27302126

[44] Steiner T, Al-Shahi Salman R, Beer R, Christensen H, Cordonnier C, Csiba L, et al. European stroke organisation (ESO) guidelines for the management of spontaneous intracerebral hemorrhage. Int J Stroke. 2014. 10.1111/ijs.12309 25156220

[45] Raval AN, Cigarroa JE, Chung MK, Diaz-Sandoval LJ, Diercks D, Piccini JP, et al. Management of Patients on non–vitamin K antagonist Oral anticoagulants in the acute care and Periprocedural setting: a scientific statement from the American Heart Association. Circulation. 2017. 10.1161/CIR.0000000000000477 PMC5404934 28167634

[46] Steiner T, Rosand J, Diringer M. Intracerebral hemorrhage associated with oral anticoagulant therapy: current practices and unresolved questions. Stroke. 2006. 10.1161/01.STR.0000196989.09900.f8 16339459

[47] Gerner ST, Kuramatsu JB, Sembill JA, Sprügel MI, Endres M, Haeusler KG, et al. Association of prothrombin complex concentrate administration and hematoma enlargement in non–vitamin K antagonist oral anticoagulant–related intracerebral hemorrhage. Ann Neurol. 2018. 10.1002/ana.25134 29314216

[48] Andrews H, Rittenhouse K, Gross B, Rogers FB. The effect of time to international normalized ratio reversal on intracranial hemorrhage evolution in patients with traumatic brain injury. J Trauma Nurs. 2017. 10.1097/JTN.0000000000000330 29117058

[49] Watson VL, Louis N, Seminara BV, Paul Muizelaar J, Alberico A. Proposal for the rapid reversal of coagulopathy in patients with nonoperative head injuries on anticoagulants and/or antiplatelet agents: a case study and literature review. Neurosurgery. 2017. 10.1093/neuros/nyx072 28368482

[50] Steffel J, Verhamme P, Potpara TS, Albaladejo P, Antz M, Desteghe L, et al. The 2018 European heart rhythm association practical guide on the use of non-vitamin K antagonist oral anticoagulants in patients with atrial fibrillation. Eur Heart J. 2018. 10.5603/KP.2018.0180 30211938

[51] Verma A, Ha ACT, Rutka JT, Verma S. What surgeons should know about non–Vitamin K oral anticoagulants a review. JAMA Surgery. 2018. 10.1001/jamasurg.2018.0374 29710221

[52] Eikelboom JW, Kozek-Langenecker S, Exadaktylos A, Batorova A, Boda Z, Christory F, et al. Emergency care of patients receiving non-vitamin K antagonist oral anticoagulants. Br J Anaesth. 2017. 10.1016/j.bja.2017.11.082 29576106

[53] Watson HG, Baglin T, Laidlaw SL, Makris M, Preston FE. A comparison of the efficacy and rate of response to oral and intravenous Vitamin K in reversal of over-anticoagulation with warfarin. Br J Haematol. 2001. 10.1046/j.1365-2141.2001.03070.x 11722425

[54] Yasaka M, Naritomi H, Minematsu K. Ischemic stroke associated with brief cessation of warfarin. Thrombosis Research. 2006. 10.1016/j.thromres.2005.08.009 16197984

[55] Lubetsky A, Hoffman R, Zimlichman R, Eldor A, Zvi J, Kostenko V, et al. Efficacy and safety of a prothrombin complex concentrate (Octaplex®) for rapid reversal of oral anticoagulation. Thromb Res. 2004. 10.1016/j.thromres.2004.04.004 15226091

[56] Hung A, Singh S, Tait RC. A prospective randomized study to determine the optimal dose of intravenous vitamin K in reversal of over-warfarinization. Br J Haematol. 2000. 10.1046/j.1365-2141.2000.02001.x 10886201

[57] Loftus CM. Anticoagulation and hemostasis in neurosurgery. 2016.

[58] Crowther MA, Wilson S. Vitamin K for the treatment of asymptomatic coagulopathy associated with Oral anticoagulant therapy. J Thromb Thrombolysis. 2003. 10.1023/B:THRO.0000014597.87575.e9 14760216

[59] Ageno W, Crowther M, Steidl L, Ultori C, Mera V, Dentali F (2002). Low dose oral vitamin K to reverse acenocoumarol-induced coagulopathy: a randomized controlled trial. Thromb Haemost.

[60] Yasaka M, Sakata T, Minematsu K, Naritomi H. Correction of INR by prothrombin complex concentrate and vitamin K in patients with warfarin related hemorrhagic complication. Thromb Res. 2002. 10.1016/s0049-3848(02)00402-4 12586128

[61] Lubetsky A, Yonath H, Olchovsky D, Loebstein R, Halkin H, Ezra D. Comparison of Oral vs intravenous Phytonadione (vitamin K1) in patients with excessive anticoagulation: a prospective randomized controlled study. Arch Intern Med. 2003. 10.1001/archinte.163.20.2469 14609783

[62] Shetty HGM, Backhouse G, Bentley DP, Routledge PA (1992). Effective reversal of warfarin-induced excessive anticoagulation with low dose vitamin K1. Thromb Haemost.

[63] Wiegele M, Schöchl H, Haushofer A, Ortler M, Leitgeb J, Kwasny O (2019). Diagnostic and therapeutic approach in adult patients with traumatic brain injury receiving oral anticoagulant therapy: an Austrian interdisciplinary consensus statement. Crit Care.

[64] Schulman S, Bijsterveld NR. Anticoagulants and Their Reversal. Transfus Med Rev. 2007. 10.1016/j.tmrv.2006.08.002 17174219

[65] Makris M, Greaves M, Phillips WS, Kitchen S, Rosendaal FR, Preston FE (1997). Emergency oral anticoagulant reversal: the relative efficacy of infusions of fresh frozen plasma and clotting factor concentrate on correction of the coagulopathy. Thromb Haemost.

[66] Woo CH, Patel N, Conell C, Rao VA, Faigeles BS, Patel MC, et al. Rapid warfarin reversal in the setting of intracranial hemorrhage: a comparison of plasma, recombinant activated factor vii, and prothrombin complex concentrate. World Neurosurgery. 2014. 10.1016/j.wneu.2012.12.002 23220122

[67] Sarode R, Milling TJ, Refaai MA, Mangione A, Schneider A, Durn BL, et al. Efficacy and safety of a 4-factor prothrombin complex concentrate in patients on vitamin K antagonists presenting with major bleeding: a randomized, plasma-controlled, phase IIIb study. Circulation. 2013. 10.1161/CIRCULATIONAHA.113.002283 PMC6701181 23935011

[68] Goldstein JN, Refaai MA, Milling TJ, Lewis B, Goldberg-Alberts R, Hug BA, et al. Four-factor prothrombin complex concentrate versus plasma for rapid vitamin K antagonist reversal in patients needing urgent surgical or invasive interventions: a phase 3b, open-label, non-inferiority, randomised trial. Lancet. 2015. 10.1016/S0140-6736(14)61685-8 PMC6633921 25728933

[69] Al-Majzoub O, Rybak E, Reardon DP, Krause P, Connors JM. Evaluation of warfarin reversal with 4-factor Prothrombin complex concentrate compared to 3-factor Prothrombin complex concentrate at a tertiary Academic Medical Center. J Emerg Med. 2016. 10.1016/j.jemermed.2015.07.024 26433428

[70] Jones CA, Ducis K, Petrozzino J, Clark E, Fung MK, Peters C, et al. Prevention of treatment-related fluid overload reduces estimated effective cost of prothrombin complex concentrate in patients requiring rapid vitamin K antagonist reversal. Expert Rev Pharmacoeconomics Outcomes Res. 2016. 10.1586/14737167.2015.1071194 26211539

[71] Sarode R, Matevosyan K, Bhagat R, Rutherford C, Madden C, Beshay JE. Rapid warfarin reversal: a 3-factor prothrombin complex concentrate and recombinant factor VIIa cocktail for intracerebral hemorrhage. J Neurosurg. 2012. 10.3171/2011.11.JNS11836 22175718

[72] Brody DL, Aiyagari V, Shackleford AM, Diringer MN. Use of recombinant factor VIIa in patients with warfarin-associated intracranial hemorrhage. Neurocrit Care. 2005. 10.1385/NCC:2:3:263 PMC2535929 16159073

[73] Aledort LM. Comparative thrombotic event incidence after infusion of recombinant factor VIIa versus factor VIII inhibitor bypass activity. J Thromb Haemost. 2004. 10.1111/j.1538-7836.2004.00944.x 15456478

[74] Butenas S, Brummel KE, Branda RF, Paradis SG, Mann KG. Mechanism of factor VIIa-dependent coagulation in hemophilia blood. Blood. 2002. 10.1182/blood.v99.3.923 11806995

[75] O’Connell K A, Wood JJ, Wise RP, Lozier JN, Braun MM. Thromboembolic adverse events after use of recombinant human coagulation factor VIIa. JAMA. 2006. 10.1001/jama.295.3.293 16418464

[76] Awad NI, Cocchio C. Activated prothrombin complex concentrates for the reversal of anticoagulant-associated coagulopathy. P T. 2013. PMC3875259 24391389

[77] Kissela BM, Eckman MH. Cost effectiveness of recombinant factor VIIa for treatment of intracerebral hemorrhage. BMC Neurol. 2008. 10.1186/1471-2377-8-17 PMC2397434 18489750

[78] Pollack CV, Reilly PA, van Ryn J, Eikelboom JW, Glund S, Bernstein RA, et al. Idarucizumab for Dabigatran reversal — full cohort analysis. N Engl J Med. 2017. 10.1056/NEJMoa1707278 28693366

[79] Connolly SJ, Milling TJ, Eikelboom JW, Gibson CM, Curnutte JT, Gold A, et al. Andexanet Alfa for Acute Major Bleeding Associated with Factor Xa Inhibitors. N Engl J Med. 2016. 10.1056/NEJMoa1607887 PMC5568772 27573206

[80] Ansell JE, Bakhru SH, Laulicht BE, Steiner SS, Grosso M, Brown K, et al. Use of PER977 to reverse the anticoagulant effect of edoxaban. N Engl J Med. 2014. 10.1056/NEJMc1411800 25371966

[81] Connolly SJ, Crowther M, Eikelboom JW, Gibson CM, Curnutte JT, Lawrence JH (2019). Full study report of andexanet alfa for bleeding associated with factor Xa inhibitors. N Engl J Med.

[82] Heidbuchel H, Verhamme P, Alings M, Antz M, Diener H-C, Hacke W, et al. Updated European heart rhythm association practical guide on the use of non-vitamin-K antagonist anticoagulants in patients with non-valvular atrial fibrillation: executive summary. Eur Heart J. 2016. 10.1093/europace/euv309 26324838

[83] Testa S, Legnani C, Tripodi A, Paoletti O, Pengo V, Abbate R, et al. Poor comparability of coagulation screening test with specific measurement in patients receiving direct oral anticoagulants: results from a multicenter/multiplatform study. J Thromb Haemost. 2016. 10.1111/jth.13486 27566988

[84] Božič-Mijovski M, Malmström RE, Malovrh P, Antovic JP, Vene N, Šinigoj P, et al. Diluted thrombin time reliably measures low to intermediate plasma dabigatran concentrations. Ann Clin Biochem. 2015. 10.1177/0004563215599795 26392631

[85] Körber MK, Langer E, Köhr M, Wernecke KDi, Korte W, Von Heymann C. In vitro and ex vivo Measurement of Prophylactic Dabigatran Concentrations with a New Ecarin-Based Thromboelastometry Test. In: Transfusion Medicine and Hemotherapy. 2017. 10.1159/000470622 PMC5425765 28503126

[86] Studt JD, Alberio L, Angelillo-Scherrer A, Asmis LM, Fontana P, Korte W, et al. Accuracy and consistency of anti-Xa activity measurement for determination of rivaroxaban plasma levels. J Thromb Haemost. 2017. 10.1111/jth.13747 28574652

[87] He L, Kochan J, Lin M, Vandell A, Brown K, Depasse F. Determination of edoxaban equivalent concentrations in human plasma by an automated anti-factor Xa chromogenic assay. Thromb Res. 2017. 10.1016/j.thromres.2017.05.005 28535438

[88] Douxfils J, Mullier F, Robert S, Chatelain C, Chatelain B, Dogné JM. Impact of dabigatran on a large panel of routine or specific coagulation assays: laboratory recommendations for monitoring of dabigatran etexilate. Thromb Haemost. 2012. 10.1160/TH11-11-0804 22438031

[89] Samama MM, Meddahi S, Samama CM. Pharmacology and laboratory testing of the oral Xa inhibitors. Clin Lab Med. 2014. 10.1016/j.cll.2014.06.009 25168939

[90] Doherty JU, Gluckman TJ, Hucker WJ, Januzzi JL, Ortel TL, Saxonhouse SJ, et al. ACC expert consensus decision pathway for Periprocedural Management of Anticoagulation in patients with Nonvalvular atrial fibrillation: a report of the American College of Cardiology Clinical Expert Consensus Document Task Force. J Am Coll Cardiol. 2017. 10.1016/j.jacc.2016.11.024 28081965

[91] Lazio BE, Simard JM. Anticoagulation in neurosurgical patients. Neurosurgery. 1999. 10.1097/00006123-199910000-00022 10515479

[92] Skardelly M, Mönch L, Roder C, Hockel K, Tatagiba MS, Ebner FH (2018). Survey of the management of perioperative bridging of anticoagulation and antiplatelet therapy in neurosurgery. Acta Neurochir.

[93] Albrecht JS, Liu X, Baumgarten M, Langenberg P, Rattinger GB, Smith GS, et al. Benefits and risks of anticoagulation resumption following traumatic brain injury. JAMA Intern Med. 2014. 10.1001/jamainternmed.2014.2534 PMC4527047 24915005

[94] Staerk L, Fosbøl EL, Lamberts M, Bonde AN, Gadsbøll K, Sindet-Pedersen C, et al. Resumption of oral anticoagulation following traumatic injury and risk of stroke and bleeding in patients with atrial fibrillation: a nationwide cohort study. Eur Heart J. 2018. 10.1093/eurheartj/ehx598 29165556

[95] Puckett Y, Zhang K, Blasingame J, Lorenzana J, Parameswaran S, Brooks SE, et al. Safest Time to Resume Oral Anticoagulation in Patients with Traumatic Brain Injury Cureus 2018;10:e2920. doi:10.7759/cureus.2920. 10.7759/cureus.2920 PMC6122643 30186725

[96] Tykocki T, Guzek K. Anticoagulation Therapy in Traumatic Brain Injury. World Neurosurgery. 2016. 10.1016/j.wneu.2016.01.063 26850974

[97] Guha D, Coyne S, Macdonald RL. Timing of the resumption of antithrombotic agents following surgical evacuation of chronic subdural hematomas: a retrospective cohort study. J Neurosurg. 2016. 10.3171/2015.2.JNS141889 26361283

[98] Ducruet AF, Grobelny BT, Zacharia BE, Hickman ZL, DeRosa PL, Anderson K, et al. The surgical management of chronic subdural hematoma. Neurosurgical Review. 2012. 10.1007/s10143-011-0349-y 21909694

[99] Chao T-F, Liu C-J, Lin Y-J, Chang S-L, Lo L-W, Hu Y-F, et al. Oral Anticoagulation in Very Elderly Patients with Atrial Fibrillation - A Nationwide Cohort Study. Circulation. 2018. 10.1161/CIRCULATIONAHA.117.031658 29490992

[100] Poon MTC, Fonville AF, Salman RAS. Long-term prognosis after intracerebral haemorrhage: systematic review and meta-analysis. J Neurol Neurosurg Psychiatry. 2014. 10.1136/jnnp-2013-306476 24262916

[101] Smith EE, Greenberg SM. Clinical diagnosis of cerebral amyloid angiopathy: validation of the Boston criteria. Curr Atheroscler Rep. 2003. 10.1007/s11883-003-0048-4 12793966

[102] Martinez-Ramirez S, Ayres A, Gurol E, Rosand J, Greenberg SM, Viswanathan A. Diagnostic value of lobar microbleeds without hemorrhagic stroke: a radiological-pathological correlation study. American Academy of Neurology Meeting. 2012.

[103] Banerjee G, Carare R, Cordonnier C, Greenberg SM, Schneider JA, Smith EE, et al. The increasing impact of cerebral amyloid angiopathy: essential new insights for clinical practice. Journal of Neurology, Neurosurgery and Psychiatry. 2017. 10.1136/jnnp-2016-314697 PMC5740546 28844070

[104] Biffi A, Kuramatsu JB, Leasure A, Kamel H, Kourkoulis C, Schwab K, et al. Oral Anticoagulation and Functional Outcome after Intracerebral Hemorrhage. Ann Neurol. 2017. 10.1002/ana.25079 PMC5730065 29028130

[105] LeRoux P, Pollack C V., Milan M, Schaefer A. Race against the clock: overcoming challenges in the management of anticoagulant-associated intracerebral hemorrhage. Journal of neurosurgery. 2014;121 Suppl:1–20. 10.3171/2014.8.paradigm 25081496

[106] Douketis JD, Spyropoulos AC, Spencer FA, Mayr M, Jaffer AK, Eckman MH, et al. Perioperative management of antithrombotic therapy. Antithrombotic therapy and prevention of thrombosis, 9th ed: American College of Chest Physicians evidence-based clinical practice guidelines. Chest. 2012;141 2 SUPPL.:e326S-e350S. 10.1378/chest.11-2298 PMC3278059 22315266

[107] Khan NR, Patel PG, Sharpe JP, Lee SL, Sorenson J. Chemical venous thromboembolism prophylaxis in neurosurgical patients: an updated systematic review and meta-analysis. J Neurosurg. 2017. 10.3171/2017.2.JNS162040 29192859

[108] Farooqui A, Hiser B, Barnes SL, Litofsky NS. Safety and efficacy of early thromboembolism chemoprophylaxis after intracranial hemorrhage from traumatic brain injury. J Neurosurg. 2013. 10.3171/2013.8.JNS13424 24053504

[109] Boeer A, Voth E, Henze T, Prange HW. Early heparin therapy in patients with spontaneous intracerebral haemorrhage. J Neurol Neurosurg Psychiatry. 1991. 10.1136/jnnp.54.5.466 PMC488553 1865215

[110] Douketis JD, Spyropoulos AC, Kaatz S, Becker RC, Caprini JA, Dunn AS, et al. BRIDGE - Perioperative Bridging Anticoagulation in Patients with Atrial Fibrillation. N Engl J Med. 2016. 10.1056/NEJMoa1501035 PMC4931686 26095867

