# Supplementary figures and images for: How do you manage ANTICOagulant therapy in neurosurgery? The ANTICO survey of the Italian Society of Neurosurgery (SINCH)
Source: BMC Neurol. 2021 Mar 3;21:98. doi: 10.1186/s12883-021-02126-7 (PMC7927258; doi:10.1186/s12883-021-02126-7)

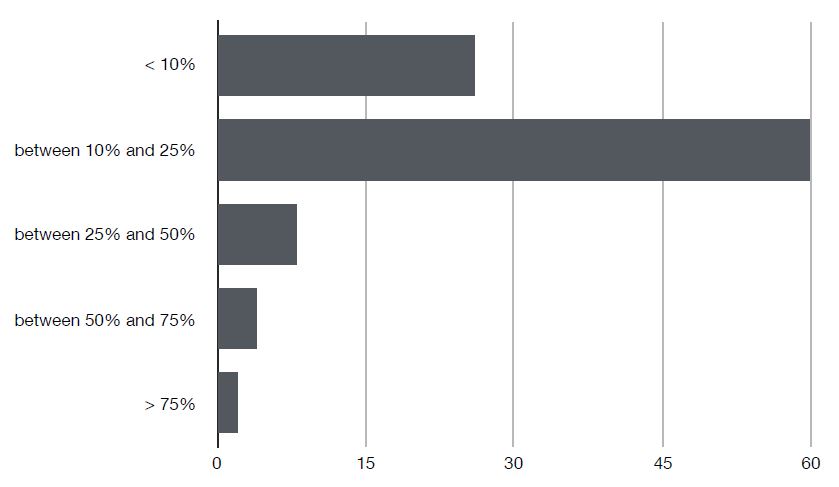

Supplement: Supplementary file 1 — Additional file 1: Supplementary Fig. 1. QUESTION 1: In the last year, indicate the approximate percentage of patients admitted in your department with “acute” neurosurgical indication. Possible answers: less than 10%, between 10 and 25%, between 25 and 50%, between 50 and 75%, more than 75%. [file 12883_2021_2126_MOESM1_ESM.jpg]

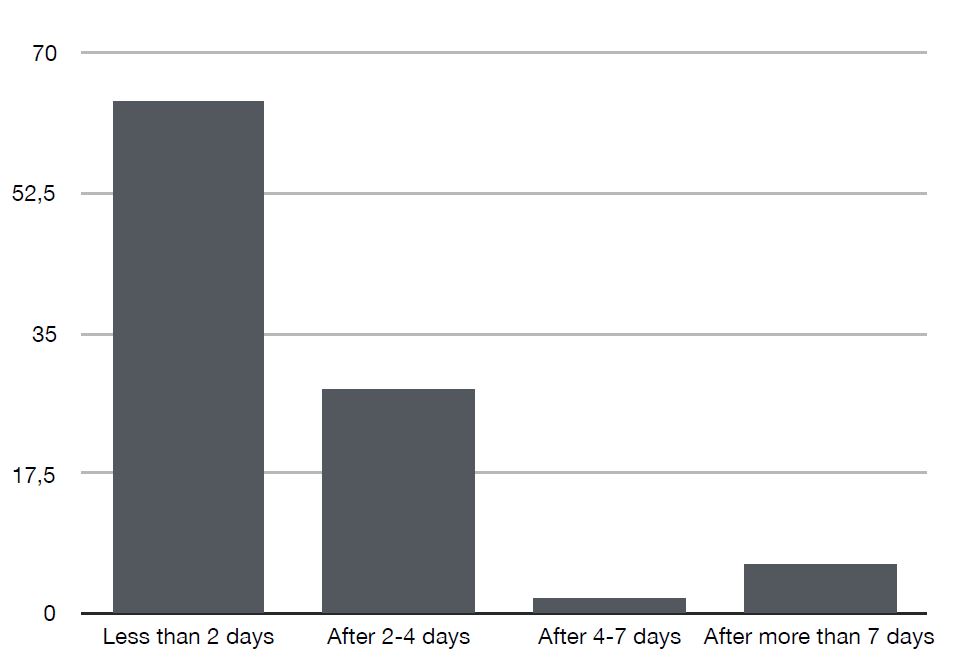

Supplement: Supplementary file 2 — Additional file 2: Supplementary Fig. 2. QUESTION 6: What is the optimal timing for initiating venous thromboembolism chemoprophylaxis after intracranial bleeding or after elective surgery? Possible answers: less than 2 days, between 2 and 4 days, between 4 and 7 days and more than 7 days [file 12883_2021_2126_MOESM2_ESM.jpg]

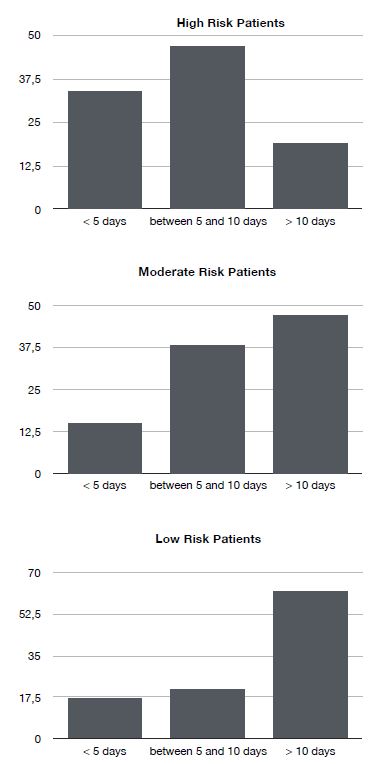

Supplement: Supplementary file 3 — Additional file 3: Supplementary Fig. 3. QUESTIONS 8, 9 and 10: In your opinion, what is the optimal timing for anti-thrombotic therapy resumption in patients at high thrombotic risk (e.g. valvular atrial fibrillation, ventricular devices), moderate thrombotic risk (e.g. non-valvular atrial fibrillation) and low-thrombotic risk (e.g. previous history of deep venous thrombosis)? [file 12883_2021_2126_MOESM3_ESM.jpg]
