# Supplementary material for: How do you manage ANTICOagulant therapy in neurosurgery? The ANTICO survey of the Italian Society of Neurosurgery (SINCH)
Source: BMC Neurol. 2021 Mar 3;21:98. doi: 10.1186/s12883-021-02126-7 (PMC7927258; doi:10.1186/s12883-021-02126-7)
Supplement: Supplementary file 4 — Additional file 4. [file 12883_2021_2126_MOESM4_ESM.docx]

| ***Question 1: In the last year, indicate the approximate percentage of patients admitted in your department with "acute" neurosurgical indication (e.g. acute/chronic epidural-subdural hematomas, ICH, SAH, Traumatic Subarachnoid Hemorrhage), in anticoagulant therapy.*** |
| --- |
| *Possible answers* |
| - *less than 10%* |
| - *between 10 and 25%* |
| - *between 25 and 50%* |
| - *between 50 and 75%* |
| - *more than 75%.* |
| ***Question 2:In the clinical scenario of the above mentioned acute neurosurgical pathologies, in which conditions do you apply a "forced" emergency reversal of anticoagulant agents?*** |
| *Possible answers:* |
| - *Only if a surgical treatment is plenned* |
| - *Also If a conservative treatment is planned* |
| ***Question 3: In the eventuality of the clinical scenario of the above mentioned “acute neurosurgical pathologies”, how do you reverse VKA patients?For every option listened, please choose among routinely, frequently, rarely and never*** |
| ***Vitamin K*** |
| - *routinely* |
| - *frequently* |
| - *rarely* |
| - *never* |
| ***Vitamin K plus Fresh Frozen Plasma*** |
| *routinely* |
| - *frequently* |
| - *rarely* |
| - *never* |
| ***Vitamin K plus Prothrombin Complex Concentrate*** |
| - *routinely* |
| - *frequently* |
| - *rarely* |
| - *never* |
| ***Prothrombin Complex Concentrate alone*** |
| - *routinely* |
| - *frequently* |
| - *rarely* |
| - *never* |
| ***Recombinant Activated Factor VII*** |
| - *routinely* |
| - *frequently* |
| - *rarely* |
| - *never* |
| ***Recombinant Activated Factor VII plus Vitamin K*** |
| - *routinely* |
| - *frequently* |
| - *rarely* |
| - *never* |
| ***Question 4: In the eventuality of the clinical scenario of the above mentioned (“acute neurosurgical pathologies”), how do you reverse DOAC patients?For every option listened, please choose among routinely, frequently, rarely and never*** |
| ***Vitamin K*** |
| - *routinely* |
| - *frequently* |
| - *rarely* |
| - *never* |
| ***Vitamin K plus Fresh Frozen Plasma*** |
| *routinely* |
| - *frequently* |
| - *rarely* |
| - *never* |
| ***Vitamin K plus Prothrombin Complex Concentrate*** |
| - *routinely* |
| - *frequently* |
| - *rarely* |
| - *never* |
| ***Prothrombin Complex Concentrate alone*** |
| - *routinely* |
| - *frequently* |
| - *rarely* |
| - *never* |
| ***Activated Prothrombin Complex Concentrate*** |
| - *routinely* |
| - *frequently* |
| - *rarely* |
| - *never* |
| ***Recombinant Activated Factor VII*** |
| - *routinely* |
| - *frequently* |
| - *rarely* |
| - *never* |
| ***Recombinant Activated Factor VII plus Vitamin K*** |
| - *routinely* |
| - *frequently* |
| - *rarely* |
| - *never* |
| ***Specific Reversal Agent (if available)*** |
| - *routinely* |
| - *frequently* |
| - *rarely* |
| - *never* |
| ***Question 5:How do you assess anticoagulant effects in patients on DOACSs with acute neurosurgical pathologies? (multiple answers possible)*** |
| - *Drug’s half-life* |
| - *Time from the last intake of the drug* |
| - *PT/aPTT* |
| - *INR* |
| - *Specific assay* |
| ***Question 6:In your opinion, what is the optimal timing for anti-thrombotic therapy resumption in patients at high thrombotic risk (e.g. valvular atrial fibrillation, ventricular devices)?*** |
| - *less than 5 days* |
| - *between 5 and 10 days* |
| - *more than 10 days* |
| ***Question 7:In your opinion, what is the optimal timing for anti-thrombotic therapy resumption in patients at moderate thrombotic risk (e.g. non-valvular atrial fibrillation)?*** |
| - *less than 5 days* |
| - *between 5 and 10 days* |
| - *more than 10 days* |
| ***Question 8:In your opinion, what is the optimal timing for anti-thrombotic therapy resumption in patients at low-thrombotic risk (e.g. previous history of deep venous thrombosis)?*** |
| - *less than 5 days* |
| - *between 5 and 10 days* |
| - *more than 10 days* |
| ***Question 9:What is the optimal timing for initiating venous thromboembolism chemoprophylaxis after intracranial bleeding or after elective surgery?*** |
| *Less than 2 days* |
| *between 2 and 4 days* |
| *between 4 and 7 days* |
| *more than 7 days* |
| ***Question 10:Do you usually ask for a cardiological evaluation for the perioperative management of anticoagulated patients?*** |
| - *Yes* |
| - *No* |
